# A novel hybrid soft computing optimization framework for dynamic economic dispatch problem of complex non-convex contiguous constrained machines

**DOI:** 10.1371/journal.pone.0261709

**Published:** 2022-01-26

**Authors:** Ijaz Ahmed, Um-E-Habiba Alvi, Abdul Basit, Tayyaba Khursheed, Alwena Alvi, Keum-Shik Hong, Muhammad Rehan

**Affiliations:** 1 Department of Electrical Engineering, Pakistan Institute of Engineering and Applied Sciences (PIEAS), Islamabad, Pakistan; 2 Department of Electrical Engineering, Mehran University of Engineering Technology (MUET), Jamshoro, Sindh, Pakistan; 3 Department of Electrical Engineering, Dawood University of Engineering and Technology (DUET), Karachi, Sindh, Pakistan; 4 Department of Management Sciences, Bahria University Karachi Campus (BUKC), Karachi, Sindh, Pakistan; 5 School of Mechanical Engineering and Department of Cogno-Mechatronics Engineering, Pusan National University, Busan, Republic of Korea; J.C. Bose University of Science and Technology, YMCA, INDIA, INDIA

## Abstract

The reformations of the electrical power sector have resulted in very dynamic and competitive market that has changed many elements of the power industry. Excessive demand of energy, depleting the fossil fuel reserves of planet and releasing the toxic air pollutant, has been causing harm to earth habitats. In this new situation, insufficiency of energy supplies, rising power generating costs, high capital cost of renewable energy equipment, environmental concerns of wind power turbines, and ever-increasing demand for electrical energy need efficient economic dispatch. The objective function in practical economic dispatch (ED) problem is nonlinear and non-convex, with restricted equality and inequality constraints, and traditional optimization methods are incapable of resolving such non-convex problems. Over the recent decade, meta-heuristic optimization approaches have acquired enormous reputation for obtaining a solution strategy for such types of ED issues. In this paper, a novel soft computing optimization technique is proposed for solving the dynamic economic dispatch problem (DEDP) of complex non-convex machines with several constraints. Our premeditated framework employs the genetic algorithm (GA) as an initial optimizer and sequential quadratic programming (SQP) for the fine tuning of the pre-optimized run of GA. The simulation analysis of GA-SQP performs well by acquiring less computational cost and finite time of execution, while providing optimal generation of powers according to the targeted power demand and load, whereas subject to valve point loading effect (VPLE) and multiple fueling option (MFO) constraints. The adequacy of the presented strategy concerning accuracy, convergence as well as reliability is verified by employing it on ten benchmark case studies, including non-convex IEEE bus system at the same time also considering VPLE of thermal power plants. The potency of designed optimization seems more robust with fast convergence rate while evaluating the hard bounded DEDP. Our suggested hybrid method GA-SQP converges to achieve the best optimal solution in a confined environment in a limited number of simulations. The simulation results demonstrate applicability and adequacy of the given hybrid schemes over conventional methods.

## Introduction

The essential purpose of the energy dispatch problem (EDP) is scheduling the electric generation of units to attain the lowest possible cost while also satisfying the constraints associated within the system. The optimal EDP solution is one of the most important factors in achieving the power system’s sustainability while focusing on low dispatch price, achieving demand-supply balance, and maintaining constraints such as valve-point loading (VPL) effects, prohibited operating zones (POZ), ramp-rate limits (RRL) and power generation limits [1–3]. For the solution of the EDP, several methods were initially developed, including lamda-iterative, gradient-based, and projection techniques [4–6]. The unmeasured supply of energy system is ceaselessly expanding and inflicting the scientists around the globe to meet challenging demand of electricity market. More than 80 percent of world’s electricity is generated through fossil fuels [7] so advocating advanced flexible soft computing optimization framework to confederate with the reduction in operation costs of large scale power plants.

It should be noted that machine-learning mechanisms are being utilized effectively in optimization and detection tasks, as observed in [8–16]. Such approaches can also be applied for the convergence analysis along with the regulation methods [17, 18]. For instance, the studies in [16, 19–23] are successful in attaining the prescribed convergence rate with real-time applicability of learning-based algorithms. Recently, a variety of literature proposed different methodologies to address economic dispatch problem. Most of these approaches, however, support convex machines with convex constraints which are easily to handle. In our work, we include highly non-convex constraints for fuel cost optimization of large bench mark test systems. The authors in [24] solved the EDP by determining optimal incremental cost of quadratic function on the basis of control theory (see [25–27]). The mismatch amongst power demand as well as active generations is handled by a feedback controller; however, this distributed optimization scheme fails if the system contains other constraints, like valve-point loading effect (VPLE) in addition to active system losses. Existing approaches for tackling the distributed optimization issues published in the recent research are unsuitable for power distribution system applications. The primary disadvantage is the huge number of communication rounds necessary, i.e., macro-iterations among the computing agents to solve one iteration of the optimization problem. Also the practical implementation of such feedback controller schemes is still an issue. The authors in [28] presented cooperative reinforcement learning algorithm for distributed generation scheme and handled the traditional complexity of the problem as the system is truly stochastic base. The computational cost and duration of the developed method are prominent, but a critical issue remains since generators are geographically scattered in different regions. Furthermore, building the communication network among the producing units is expensive, and maintaining such a network necessitates specialised staff. Extra energy capital cost is predicted as a result of the interplay between energy storage devices and producing units.

The algorithm in [29] proposed a meta-heuristic approach to solve the dynamic energy dispatch problem (DEDP) along with emission objective function. Incorporating wind energy into the combined objective function leads in lower generating costs to meet the system restrictions; nevertheless, such a system is only feasible in locations with high wind profiles. The cost of a wind turbine system is also still prohibitively expensive and the approach has the ecological issues. The authors in [30] addressed the heat generation scheduling and economic generation by validating the requirements of European commission for climate and energy policy. The efficacy of soft computing algorithms is presented in terms of cost and strict environment policies, suggested by the regulatory authorities. The performance and robustness of computing algorithms for thermo-economic sources is discussed in the article [31], and total yearly cost is minimized by fixed point iterative algorithm. The work of [32] developed EDP for nonlinear convex cost model for micro-grids to derive optimal condition for long duration of cell’s life. The authors in [33] proposed genetic and mixed integer programming for energy management system to derive optimal condition for generating units to handled unit commitment problem. They also derived the Li-ion aging model, based on event driven approach for solving the combined EDP problem. Similar work is reported in [34], where the U.S. National Renewable Energy Laboratory’s REopt model is used to optimize the distributed solar energy cost for residential buildings. While using load demand management (LDM) on 30,000 electric vehicles (EVs) during crest shaving and valley filling (CSVF) regions, the authors proposed orthogonal particle swarm optimization (OPSO) for multi-objective problem, namely dynamic economic emission dispatch, and simultaneously solved it under several practical equality and inequality operating power constraints. The method gives crucial results concerning the future functioning of PGSs when using the LDM approach on a large-scale penetration of EVs in smart cities into a sustainable environment [35]. The work in [36] presented schemes in order to solve DEDP on the basis of consensus theory. The approach predicts the stability of the designed controller on the basis of unidirectional communication among agents for a connected topology. The algorithm convergence to optimal condition is derived for IEEE-30, 57 and 300 bus systems. The authors in [37] presented a heuristic dynamic relaxation approach for dealing with the complicated constraints of the dynamic economic emission dispatch model for the wind-solar-hydro generation under trade-able green certificates. To model the random behaviour of wind, solar, and hydro power, Weibull, Beta, and Gumbel distributions are employed. Reduced carbon emissions are an essential global objective, therefore, DEDP along with renewable energy resources is studied in [38–41].

The performance of evolutionary algorithms for EDP is surveyed in the article [42]. The enactment of artificial bee colony algorithm for less computational time and also optimal cost for EDP is studied in article [43]. The losses constraint is included to show the potency of the presented approach. The algorithm in [44] handled the uncertainty constraint in EDP by using firefly and point estimate methods. The cost is minimized by incorporating energy market prices, supply demand and inclusion of renewable energy sources. Nelder–Mead algorithm is presented in [45] to address the EDP with generator constraints and transmission losses. In electrical networks, different plants feed multiple loads, and this scenario is tackled in [46] through evolutionary particle swarm optimization to determine the optimal dispatch problem by including spinning reserve constraints and failure of any generating unit. The authors in [47] presented genetic algorithm (GA) and sequential quadratic programming (SQP) optimization techniques for DELTA design configuration of haptic devices. The work of [48] used TLBO optimization for renewable sources to ensure a hybrid economic design that includes the objective function of all sources and optimized them as a single unit. The optimal condition from TLBO shows dominance among other listed techniques but the overall complexity of the algorithm is increased, and it depicts longer convergence time to obtain a reasonable solution. The suitability and practicability of meta-heuristic techniques to solve complex engineering optimization problems with multiplex restriction can be perceived in [49–55].

The primary concern of authors in this paper is to address the DEDP of non-convex machines for delivering a substitute solution by considering modern meta-heuristic techniques. The proposed approach considers GA as an initial search, followed by SQP for the fine tinning, by considering several system constraints. Comparing to the previous literature, our contribution is to deal with the complex problem based on soft computing framework while including several real-world constraints. The selection of machine is both convex and non-convex, and the designed algorithm provides confrontation for both systems. The outcomes of designed algorithm is faster convergence with finite time, best optimal conditions via handling hard bounded constraints, and less computational time and cost. The performance indexes of the designed approach addressed the generation cost considering practical constraints such as line losses, generation capacity, and valve-point loading effect, prohibited zones and multi-fueling option on standard IEEE bus systems. The major contributions in this work are outlined as follow:

1. For the above listed literature research gap, a convex and non-convex cost model along with complex constraints in the form of single objective function is reformulated to attain an optimal solution of the cost and power.
2. A GA-SQP approach is presented for DEPD through which the optimal selection of output powers of convex and non-convex machines reduce the total variable production cost thus improving the efficiency of plant and its life span.
3. The approach considers several constraints, like VPL effects, POZ, RRL and power generation limits. To ensure the system security and to avoid system contingencies, the power demand of network is fulfilled efficiently by the proposed GA-SQP computing framework. The efficacy of presented methodology is tested for both type of machines by including all constraints.
4. The selection of fuel type (MFO) with respect to its market price improves the unit cost of generating machines, which has been considered in the presented study compared to the existing studies.
5. The optimal generation and less consumption of fuel also play role in environmental aspects and regulatory institutions of emission as a result protecting environment.
6. The proposed GA-SQP approach has been compared with the existing studies of [49, 56–59]. It was observed that the proposed approach for convex and non-convex machines provides better optimal cost in a small time, compared with these methods.

The rest of article consists of problem formulation of DEDP in Section 2. Section 3 provides the proposed GA-SQP framework of optimization and parameter settings for the listed problem. Simulation results, optimizer analysis, and machines details are listed in Section 4. Finally, Section 5 provides brief discussion and conclusions.

## Problem formulation of DEDP

The aim of DEDP is to allocate or assign optimal real generated powers to generators such that the cost involving in generation will be finest while satisfying the system associated constraints.

### Convex fuel cost objective function

The convex fuel cost objective function of *n* generating units on the basis of fuel cost is given as (see for instance [60])

$$\begin{array}{c} F_{Cost}=\sum_{i=1}^{n}F_{i}(P_{Gi})=\sum_{i=1}^{n}a_{i}P_{Gi}^{2}+b_{i}P_{Gi}+c_{i}, \end{array}$$ (1)

where *F*_*i*_(*P*_*Gi*_) is the cost of fuel of *i*^*th*^ generating unit. *a*_*i*_, *b*_*i*_, and *c*_*i*_ are the coefficients of fuel cost of thermal plant. $P_{G_{i}}$ is the total active power generation in MW.

### Non-convex fuel cost objective function

**Fig 1:**
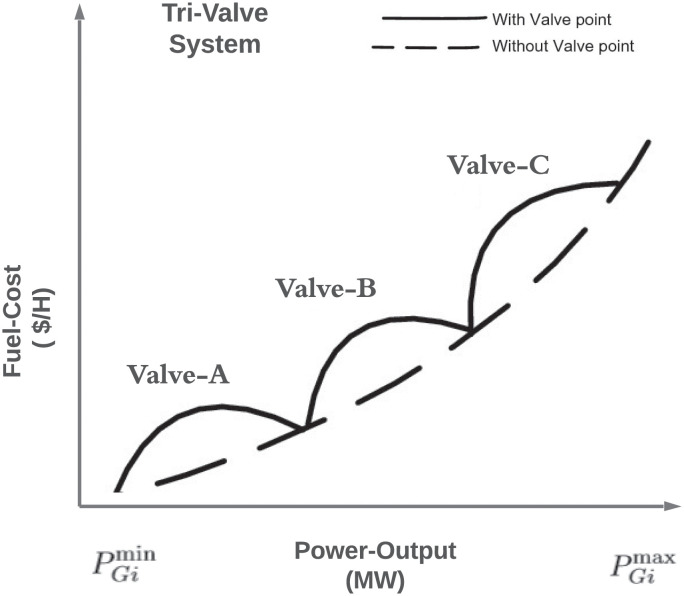
Cost function with VPLE. The figure represents curve of fuel-cost for non-convex machine.

The objective function mentioned in Eq (1) becomes non-convex due to VPLE of generating units. Addition of VPLE results into highly nonlinear accumulating ripple effects in the fuel-cost curve. Fig 1 shows the fuel-cost curve of three-valves non-convex machines. Due to VPLE, the cost function of a generating unit becomes highly non-convex, with several local sub-optimal solutions, which cannot be handled with conventional methods. The cost function due to VPLE can be expressed as in Eq (2).

$$\begin{array}{c} F_{Cost}=\sum_{i=1}^{n}F_{i}(P_{Gi})=\sum_{i=1}^{n}a_{i}P_{Gi}^{2}+b_{i}P_{Gi}+c_{i}+|e_{i}\;\text{sin}(f_{i}\left(P_{Gi}^{\text{min}}-P_{Gi}\right)|, \end{array}$$ (2)

where $P_{Gi}^{\text{min}}$ is the lower limit of generating capacity of *i*^*th*^ unit. *f*_*i*_ and *e*_*i*_ are the VPLE coefficients.

### Power demand and losses constraints

Usually, the loads are connected far from generation sites by means of transmission lines. The committed units’ active power should be equivalent to the demand and the power losses due to transmission system. The power demand loss constraint can be written as follow.

$$\begin{array}{c} \sum_{i=1}^{n}P_{Gi}=P_{demand}+P_{TLL}, \end{array}$$ (3)

where power losses in transmission lines are represented by *P*_*TLL*_ and can be expressed as follow.

$$\begin{array}{c} P_{TLL}=\sum_{i=1}^{n}\sum_{j=1}^{n}P_{i}B_{ij}P_{j}, \end{array}$$ (4)

where *P*_*i*_ is the total active power, *P*_*j*_ is the total reactive power and *B* represents the loss coefficient matrix. The elements *β*_*ij*_ of matrix

$$\begin{array}{c} P_{i}=P_{Gi}-P_{demand}-P_{TLL}, \end{array}$$ (5)

$$\begin{array}{c} \beta_{ij}=\frac{\vartheta_{ij}\;\text{cos}\;(\theta_{i}-\theta_{j})}{|W_{i}||W_{j}|\;\text{cos}\;\theta_{i}\;\text{cos}\;\theta_{j}}. \end{array}$$ (6)

where |*W*_*i*_| and |*W*_*j*_| represents the active and reactive voltages of *i*^*th*^ and *j*^*th*^ buses and *θ*_*i*_,*θ*_*j*_ indicates the phase angles between *i*^*th*^ and *j*^*th*^ buses, respectively.

### Inequality constraints

Inequality constraints are machine hardware limitations such as generation capacity and the ramp limits. Based on the generation capacities, the expression for capacity limits and ramp limits are as follow.

$$\begin{array}{c} P_{Gi}^{\text{min}}\leq P_{Gi}\leq P_{Gi}^{\text{max}}, \end{array}$$ (7)

$$\begin{array}{c} \text{max}\left\{P_{Gi}^{\text{min}},P_{Gi}^{o}-DR_{i}\right\}\leq P_{Gi}\leq \text{min}\left\{P_{Gi}^{\text{max}},P_{Gi}^{o}-UR_{i}\right\}. \end{array}$$ (8)

In the above expression $P_{Gi}^{o}$ is the previous generating point, *DR*_*i*_ the down ramp limit, and *UR*_*i*_ represents the upper ramp limit of committed generating units.

### Prohibited generating zones

Generation machines have some practical limitation, and they must be operated under these hardware thresholds, for instance, due to boiler pumps and rotor assembly mechanical governing systems. These threshold regions are referred as prohibited or forbidden regions. The mathematical model of these machines can be written as

$$\begin{array}{c} P_{Gi}=\{\begin{array}{c} P_{Gi}^{\text{min}}\leq P_{Gi}\leq P_{Gi}^{NoLoad},\\ P_{GiK-1}^{U}\leq P_{Gi}\leq P_{GiK}^{NoLoad},\\ P_{GiZI}^{U}\leq P_{Gi}\leq P_{Gi}^{MAX}, \end{array} \end{array}$$ (9)

where $P_{Gi}^{NoLoad}$ is power generated at no load, *P*_*GiK*_ is power generated at zone *K*.

### Objective function with multiple fueling option

One of the significant consideration for DEDP is multiple fueling option (MFO). The cost curve of thermal plants is based on type of fuel used in operation. Thermal units with MFO consist of different cost curves proportional to the price of fuel used. In economic operation, the lowest possible curve should be selected for optimal operations of units. Mathematically, convex fuel cost objective function with MFO of diesel and gas can be represented as follow:

$$\begin{array}{c} \begin{array}{c} F_{Cost}=\{\begin{array}{c} \sum_{i=1}^{N}F_{ij}\left(P_{G_{i}}\right)=\sum_{i=1}^{N}a_{ij}{P_{G_{i}}}^{2}+b_{ij}P_{G_{i}}+c_{ij},\\ ‥\;‥\;‥\;‥\;‥\\ ‥\;‥\;‥\;‥\;‥\\ ‥\;‥\;‥\;‥\;‥\\ \sum_{i=1}^{N}F_{ik}\left(P_{G_{i}}\right)=\sum_{i=1}^{N}a_{ik}{P_{G_{i}}}^{2}+b_{ik}P_{G_{i}}+c_{ik}, \end{array} \end{array} \end{array}$$ (10)

where *j* and *k* represents type of fuels used by thermal plants.

## Proposed optimizer framework

The perfunctory introduction of soft computing framework for DEDP is described along with procedural steps and flowcharts.

### Genetic algorithm (GA)

It is a population-based stochastic algorithm used by scientists to solve optimization problems with hard constraints [61]. GA is considered as a family of computational models that encodes a specific problem on a data based on chromosomes alike data structures and recombination operators are applied to perceive critical information of the problem [62]. GAs are efficient approaches for dealing with large-scale design optimization problems. It has undergone various changes to allow them to solve challenges more quickly, easily, and consistently. GA is a robust algorithm which is advantageous to solve complex design problems related to optimization in a number of applications. Design optimization issues can be classified in a variety of ways. One way to categorize them is into two groups that are functional and combinatorial optimization. The objective function in functional optimization is typically expressed as a continuous or piece-wise continuous function of the design parameters [63]. It is basically a search engine and a technique for optimizing designs. It was first used to model natural evolutionary development in a computing environment. GA is a set of different solutions (populations). Each population solution contains a variety of entities, vectors, and chromosomes. Individuals are then chosen using GA’s selection operator depending on their degree of conformity or usefulness, as well as objective feature [64].

The crossover and mutation processes are repeated before the stopping conditions are met. Since new chromosomes produced after crossover is subject to mutation, the likelihood of being trapped on a local minimum is reduced [65]. GA is a population-based strategy with extremely successful results in deciding the universal best since it generates many optimal solutions. In addition to populations, researchers have developed GA forms with variations in generating new individuals [66, 67]. For problems involving single-objective and multi-objective optimization, this approach, as well as variants of it, tends to produce excellent results. In the fields of industry, research, and engineering, it is widely used. GA is a stochastic model that is also robust to local maxim and minim. The only limitation this GA has is the high computation cost and time.

### Sequential quadratic programming (SQP)

Solving quadratic sub-problems is among the major powerful techniques for non-linearity constrained optimization. Sequential quadratic programming (SQP) also known as the successive quadratic programming has been used for the solution of nonlinear problems. It is an iterative method for constrained nonlinear optimization. SQP works in both trust-region frameworks as well as line search. It is also used on the complex mathematical problems in which the constraints and objective functions are twice differentials. Seeking a step away from the current point by minimising, a quadratic model of the problem makes SQP a generalisation of Newton’s method for unconstrained optimization. SQP methods illustrate their strength while solving issues with large non-linearity in constraints, as opposed to linearly constrained Lagrangian methods, which are efficient when the majority of the constraints are linear [68].

### Proposed GA-SQP approach for DEDP

Now the aim of study is to design the formulation and procedure of soft computing frameworks for DEDP. In comparison to the closely related work [47], there are vibrant differences of proposed methodology. Firstly, we have different selection of parameters for GA and SQP as provided in Table 1. It can be seen that selection of fitness and tolerance limits are set to an adequate level for attaining best outputs weights to ensure genuine convergence of solution. Secondly, these selections of parameters also empower the framework to use over large scale complex optimization problems consisting hard bounds limits of VPLE, POZ and MFO. Thirdly, the fitness evaluation limits of objective function is increased to demonstrate the practicability and convergence to best optimum candidate solutions. Possessing these distinct feature, the presented framework design acquires best optimum cost of DEDP with faster convergence rate while satisfying the all associated complex constraints.

**Table 1:** Parameter setting/values using GA and SQP.

GA		SQP	
Parameters	Settings	Parameters	Settings
Generations	500	Max Iteration	1000
Population Size	360	Max Function Evaluation	50000
Tolerance Function	1e-30	TolFun	1e-18
Stall Generation Limit	200	TolCon	1e-18
Fitness Limit	1e-30	TolX	1e-12
Population Initialization Range	[-1;1]	Others	Default

Table 1 consists of parameters and setting values for GA and SQP both.

**Fig 2:**
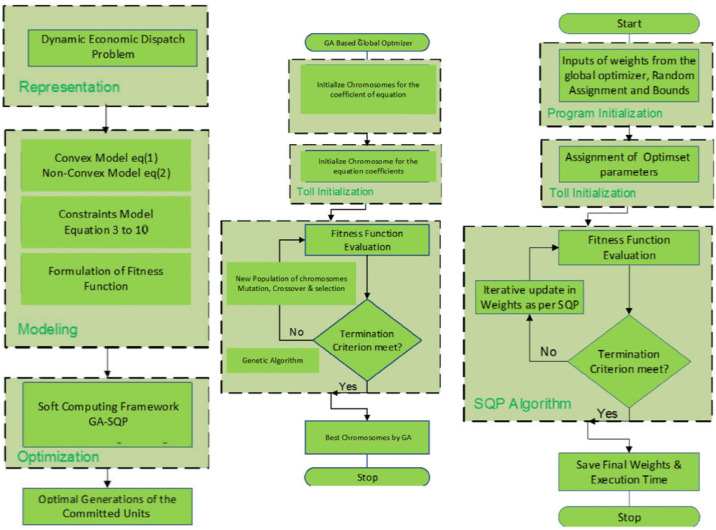
Soft computing optimizer framework. It represents three sections of flowcharts. The first flow chart describes the representation of dynamic EDP, its modeling and optimization which provides optimal generation of units. The second flow chart of Fig 2. elaborates GA based global optimizer, toll initialization, the evaluation of fitness value and finally getting the best chromosomes by GA. The third flow chart of Fig 2. represents the program initialization, toll initialization of assigning optimum parameters, SQP algorithm and then saving final weights and the execution time.

The proposed scheme is shown in Fig 2, including the DEEP along with constraints equations. Modeling is performed for two type of machines, namely convex and non-convex scenarios, and the associated constraints are also shown. For our framework, we choose GA as global optimizer with non-linear iterative programming as a local optimizer. It is efficient in terms of accuracy, convergence to provide successful solutions for complex practical world problems, for example ten systems with 100 machines.

The designed computing frame work is divided into three sections, as seen in Fig 2. First one is modeling, here we reformulate both convex and non-convex models of DEDP along with system constraints equations from (3) to (10). The fitness function for the framework is also modeled to check the convergence and diversity of the given objective function and constraints. This fitness function is used by the optimizer framework to obtain the best candidate solution for DEDP problem. The framework is composed of into two segments GA as global search technique and SQP for local search technique. First of all, the objective function through the fitness function is evaluated by GA and results are stored. Again the SQP evaluates the provided problem up to a certain level as defined in algorithm (Section 3). These results from the two frameworks are then loaded into hybrid optimization scheme. The hybrid algorithm has edge in terms of its reliability and effectiveness. The best optimal results are then obtained to show the efficacy and computational efficiency of the proposed scheme. The following is the structure of algorithm for optimising DEDP in the form of logical steps using a hybrid computational technique.

#### Logical steps of framework

*Step 1*: *M* random populations are generated which are equal to the total committed units in DEEP operation. Generally, the representation of *i*^*th*^ agent is narrated by *J*_*i*_ = [*J*_*i*1_, *J*_*i*2_, …*J*_*iN*_],*J*_*ij*_ ∈ ℜ: *L*_*b*_ ≤ *J*_*ij*_ ≤ *U*_*b*_ where *U*_*b*_ is upper bound and *L*_*b*_ is lower bound of both convex and non-convex machines.

*Step 2*: This step involves the assignment of the general parameter setting of GA algorithm used in optimization framework as shown in Table 1.

*Step 3*: This step involves evaluating fitness function by acquiring the fitness of each agent in the form of mean square error as given below.

$$\begin{array}{c} ℑ=\frac{\sum_{j=1}^{N}({\left|F_{op}^{j}-\overset{\circ_{op}^{j}}{F}\right|}^{2})}{N}, \end{array}$$ (11)

where $\overset{\circ_{op}^{j}}{F}$ is the operating cost of *j*^*th*^ committed units.

*Step 4*: It is the termination criterion step, and algorithm is terminated on the following criterion.

- Fitness value is achieved as per definition.
- All defined generations are executed.
- Tolerance function value achieved.
- All optimization functions are evaluated.

If all above conditions are achieved, stop otherwise run again.

*Step 5*: The best generated solutions are provided as input to the final design of SQP for further refinement, random assignments with problem bounds according to parameter setting provided in Table 1.

*Step 6*: This step involves the assignment of optimist parameter known as toll initialization, and step size is also monitored in this step.

*Step 7*: In this step the fitness function is evaluated according to the problem bounds with tuned tolerances setting.

*Step 8*: If convergence is achieved and termination criterion is attained then final weights with execution time are saved, otherwise run again.

## Simulations and results

In this section, MATLAB simulations for convex and non-convex objective functions for DEDP have been performed. All constraints such as transmission losses, power balance constraint, capacity constraint, and ramp limits were employed. The VPLE and MFO are also taken into account to demonstrate the efficacy of our designed soft computing framework. The parameter settings for the framework is shown in Table 1. The procedure sequence is defined as a series of logical steps. Ten case studies composed of 100 machines with highly hard constraints are selected as test systems. Required time analysis for this work is carried on Intel celeron (R) N2940 CPU @ 1.83GHz, 4.0 GB of RAM with MATLAB version R2017b.

It is actually noticeable that for a hybrid framework that the level of accuracy is dominated as compared to separate local and global techniques. The computational budget for the hybrid approach can be significantly high as compared to the local search methodologies; however, this factor can be over-shed as the accuracy and constraint handling of hybrid schemes is more versatile as compared to the local ones. GA-SQP has a better likelihood of finding a global optimum than other algorithms. In practice, GA-SQP may reach a global optimum in a limited computational time in the nonlinear or multi-constrained situations that can be enhanced by hybridising with other methods. Another reason for the lower computational cost for the hybrid method can be the operational architecture of GA-SQP, which provides many options and allows the method to quickly converge on a single viable solution.

The investigated problem may be divided into different groups, based on the nature of the fuel cost functions and constraints. In one category, DEDP problems with non-smooth cost functions as a result of taking VPL and MFO into account can be accounted. For such non-convex machines, the characteristics data of units has been provided in [59, 60, 69]. The optimal cost obtained with computational time is provided. The second category includes convex constraints and fuel cost coefficient, and data is provided in [50, 59, 60, 69, 70].

We cannot relay on the value of local search methods because they often stuck on local minima due to poor initial guess. Another point to consider is that our designed framework provides consistency and stability with finer convergence rate with increase in number of machines and constraints of DEDP.

### Case Study-I

This case study is composed of three non-convex machines with total load demand 850MW. The reference data along with fuel cost and VPLE coefficients are shown in Table 2, and optimal results shown in Table 3.

**Table 2:** Three unit system for Case Study-I.

Unit No.	a	b	c	e	f	*P*_*min*_	*P*_*max*_
CU-1	0.0016	7.9200	561	300	0.0315	100	600
CU-2	0.0019	7.8500	310	200	0.0420	100	400
CU-3	0.0048	7.9700	78	150	0.0630	50	200

**Table 3:** Optimal generations, cost, and time for Case Study-I.

Computing Framework	CU-1	CU-2	CU-3	Total Generation	Best Cost	Best Time
GA	337.0318	326.2318	186.7363	850MW	8431.4	64.6
SQP	415.7894	289.4736	144.7368	850MW	8596.6	0.077
GA-SQP	299.77	350.22	200	850MW	8421.1	0.0520

**Fig 3:**
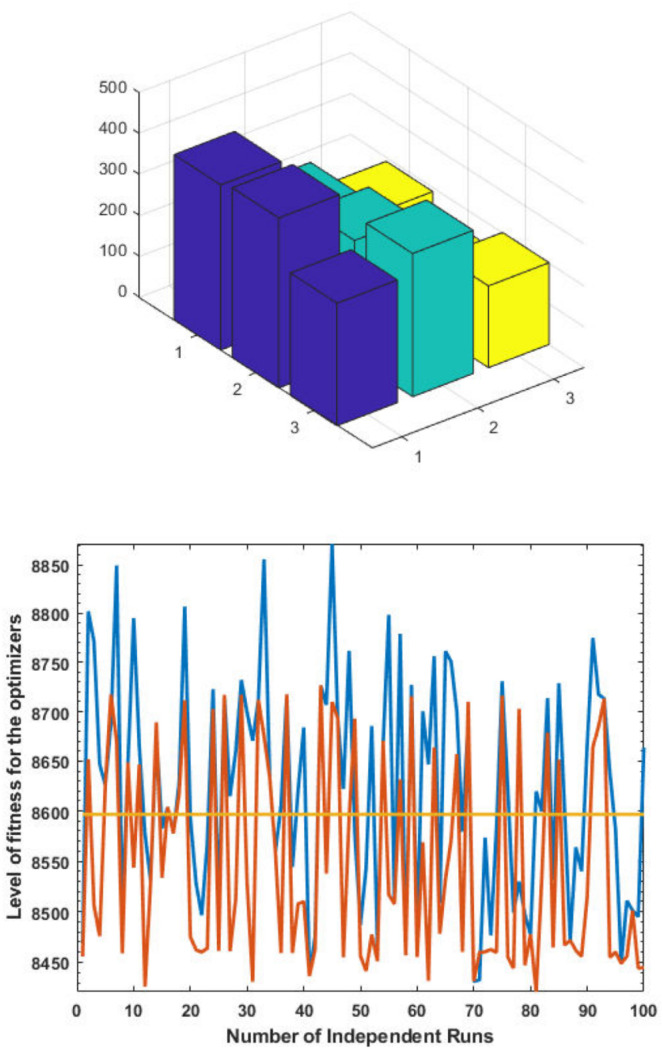
Optimizer response against three non-convex machine. A. Power output B. Fitness level.

The top fittest designed parameters that are actually the machine power generation values and are depicted in Fig 3A at semi-log scale in order to increase the size of the small effects in power values. The heights of bars indicate the generations. The axis with variable color for bars is used demonstrate different generators. The remaining axis is used to demonstrate results of GA, SQP and GA-SQP using labels 1, 2, and 3, respectively. The designed framework is based on heuristic computation so the single optimal result cannot justify the accuracy of scheme. The effectiveness of fitness, provided in Eq (11), is depicted in Fig 3B, representing the result of 100 independent run of iteration rather than single best result. The colouring scheme used by fitness level is brown, blue and golden for GA, SQP and GA-SQP respectively throughout the manuscript. The optimal cost obtained by GA-SQP for Case Study-I is 8221.1 which is better as compared to the other heuristic technique [56] which was 8231.5. By comparing the fuel cost, it is evident that our design scheme obtains the optimal result while satisfying all constraints appropriately.

### Case Study-II

**Fig 4:**
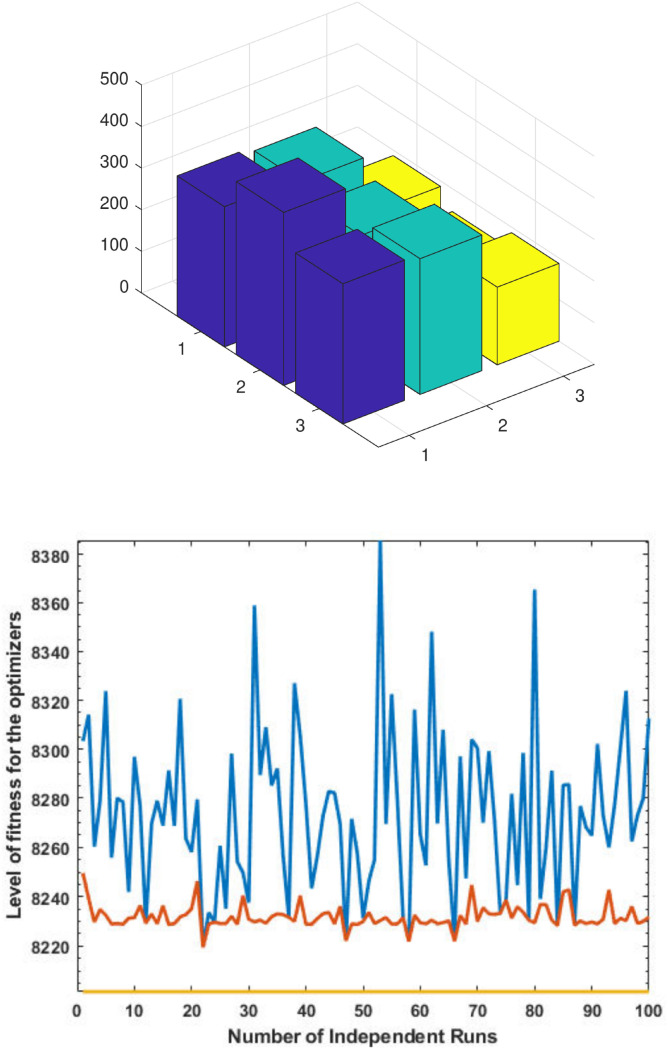
Optimizer response against three convex machines. A. Power Output B. Fitness Level.

Now we consider the application of the proposed method over convex machines and consider three convex machines with total load demand 850MW (please see Table 4). The obtained optimal results by using the GA, SQP and the proposed GA-SQP methods are shown in Table 5. Fig 4A shows the generations of three machines for three algorithms at semi-log scale. Table 5 summarizes the results of the proposed GA-SQP approach for solving the fuel cost minimization problem independently. In comparison to [57], where the cost of fuel was 8232.93, the GA-SQP approach reduces the cost to 8219.5. The effectiveness and performance of iterative procedure of fitness is plotted in Fig 4B. Hence, the proposed method is applicable to small-scale convex and non-convex machines.

**Table 4:** Three unit system for Case Study-II.

Unit No.	a	b	c	Pmin	Pmax
CU-1	0.0014	7.2000	561	100	600
CU-2	0.0019	7.8500	310	100	400
CU-3	0.0048	7.8500	78	50	200

**Table 5:** Optimal generations, cost, and time for Case Study-II.

Computing Framework	CU-1	CU-2	CU-3	Total Generation	Best Cost	Best Time
GA	404.02	257.27	188.69	850MW	8219.5	73.4
SQP	415.78	289.47	144.73	850MW	8201.5	0.122
GA-SQP	299.77	350.22	200	850MW	8219.5	0.233

### Case Study-III

A more complex system with convex machines (please see Table 6) is considered herein, which consists of six convex machines with total load demand of 283.4MW. The reference data is available at [57] along with fuel cost coefficients. Results for solving the fuel cost minimization independently using the GA-SQP approach are shown in Table 7. GA-SQP reduces the fuel cost minimization costs to 171132.9 when compared to [58], which was 191739.7.

**Table 6:** Six unit system for Case Study-III.

a	b	c	e	f
0.0076	192.6990	387.8500	0	0
0.0084	211.9690	441.6200	0	0
0.0052	219.1960	422.5700	0	0
0.0014	201.9830	552.5000	0	0
0.0015	212.1810	557.7500	0	0
0.0018	191.5280	562.1800	0	0
0.0019	210.6810	568.3900	0	0
0.0011	199.1380	682.9300	0	0
0.0012	199.8020	741.2200	0	0
0.0009	212.3520	617.8300	0	0
0.0010	210.4870	674.6100	0	0

**Table 7:** Optimal generations, cost, and time for Case Study-III.

Computing Framework	CU-1	CU-2	CU-3	CU-4	CU-5	CU-6	Total Generation	Best Cost	Best Time
GA	78.68	66.56	44.23	34.75	28.99	30.16	280MW	353004.5	99.6
SQP	128.49	51.39	33.31	23.08	20.46	26.65	280MW	307729.3	0.0143
GA-SQP	71.24	65.59	46.26	33.43	29.45	37.39	280MW	171132.9	22.003

**Fig 5:**
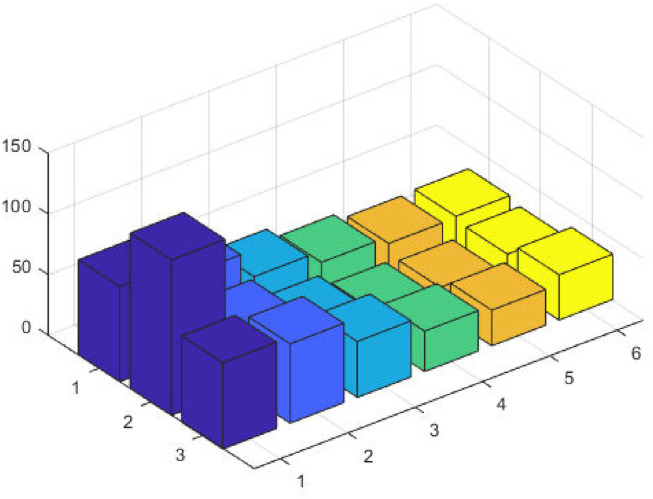
Optimizer power output response against six convex machines.

**Fig 6:**
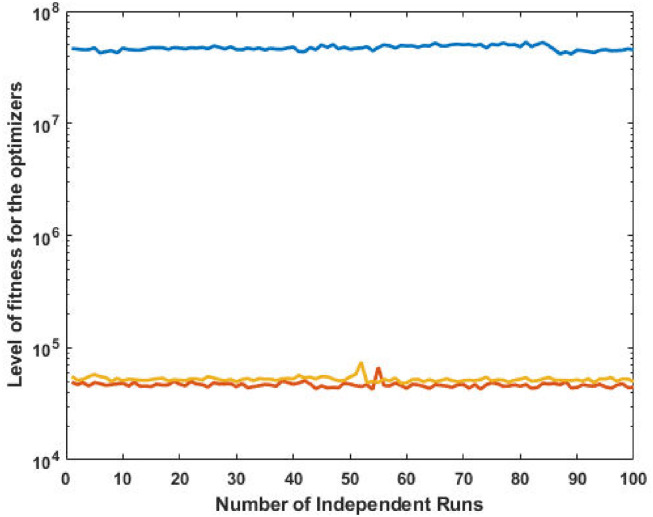
Optimizer fitness level response against six convex machines.

It is observed that the proposed GA-SQP approach performs well in optimizing the total generation cost. Fig 5 at semi-log scale demonstrates the power values for all generators. The effectiveness and performance of iterative procedure of fitness is plotted in Fig 6.

### Case Study-IV

Now we check the validity over six non-convex machines with total load demand 1800MW. The reference data along with fuel cost and VPLE coefficients is shown in Table 8 and is provided in [58], and the optimal results using our approach are shown in Table 9. Again, our approach performs well with a small time for computation to reduce the total generation cost. As shown in Table 9, the GA-SQP approach is capable of generating better solutions for the single objective function of fuel cost when compared to the conventional approach. When compared to [58], the GA-SQP reduces fuel cost minimization costs to 42442, for a total reduction of 661.16 when compared to [58, 69].

**Table 8:** Six units system for Case Study-IV.

a	b	c	e	f
0.0016	2.0000	150	50	0.0630
0.0100	2.5000	25	40	0.0980
0.0625	1.0000	0	0	0
0.0083	3.2500	0	0	0
0.0250	3.0000	0	0	0
0.0250	3.0000	0	0	0

**Table 9:** Optimal generations, cost and time for Case Study-IV.

Computing Framework	CU-1	CU-2	CU-3	CU-4	CU-5	CU-6	Total Generation	Best Cost	Best Time
GA	314.71	317.76	294.85	282.49	294.28	295.89	1800 MW	41438	77.65
SQP	299.99	299.99	299.99	299.99	299.99	299.99	1800 MW	48162	0.061
GA-SQP	265.93	302.07	319.19	313.43	316.61	282.74	1800 MW	42442	0.062

**Fig 7:**
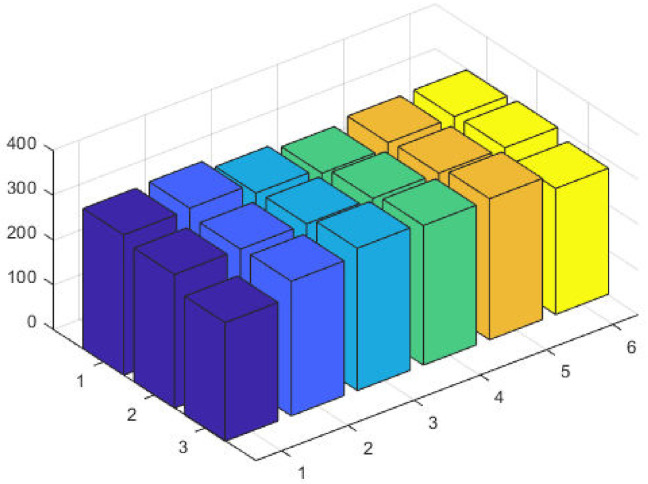
Optimizer power output response against six non-convex machines.

**Fig 8:**
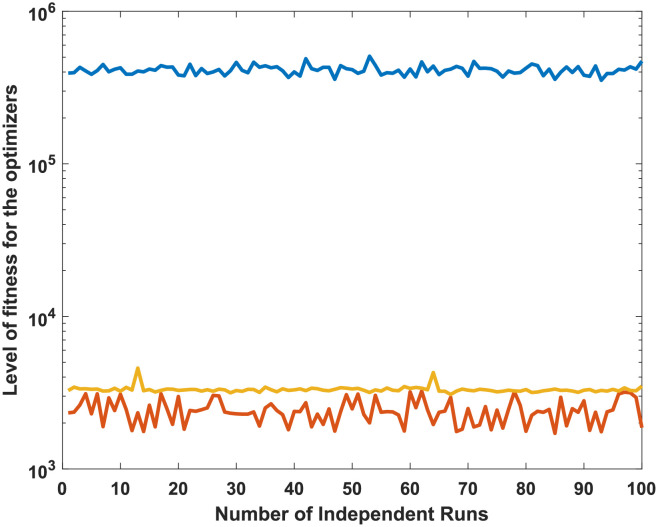
Optimizer fitness level response against six non-convex machines.

Fig 7 demonstrates the corresponding power values, and the performance of iterative procedure of fitness is plotted in Fig 8. By comparing cost from [58], our design scheme obtains optimal result while satisfying all constraints appropriately.

### Case Study-V

In Case Study-V, we consider a large system of eleven convex machines with total load demand 2500MW, the reference data of fuel cost coefficients is shown in Table 10. The optimal results are shown in Table 11. by application of the proposed method. The fuel cost savings obtained for this test system using the proposed GA-SQP are 551850, as shown in Table 11. On the other hand, the fuel cost of [59] is 570120, demonstrating the efficacy of the suggested method in achieving the lower possible cost.

**Table 10:** Eleven unit system for Case Study-V.

a	b	c	e	f
0.0076	192.6990	387.8500	0	0
0.0084	211.9690	441.6200	0	0
0.0052	219.1960	422.5700	0	0
0.0014	201.9830	552.5000	0	0
0.0015	212.1810	557.7500	0	0
0.0018	191.5280	562.1800	0	0
0.0019	210.6810	568.3900	0	0
0.0011	199.1380	682.9300	0	0
0.0012	199.8020	741.2200	0	0
0.0009	212.3520	617.8300	0	0
0.0010	210.4870	674.6100	0	0

**Table 11:** Optimal generations, cost, and time for Case Study-V.

Computing Framework	GA	SQP	GA-SQP
CU-1	177.8	166.0	182.5
CU-2	208.9	140.6	203.1
CU-3	205.4	166.0	169.0
CU-4	244.7	212.3	224.2
CU-5	162.1	140.6	187.4
CU-6	192.9	212.3	249.2
CU-7	185.1	143.7	164.4
CU-8	245.8	325.3	290.3
CU-9	299.0	325.3	235.0
CU-10	343.5	332.1	277.4
CU-11	234.2	335.3	317.1
Total Generation	2500MW	2500MW	2500MW
Best Cost	760350	576660	551850
Best Time	13.8	0.0901	0.0605

**Fig 9:**
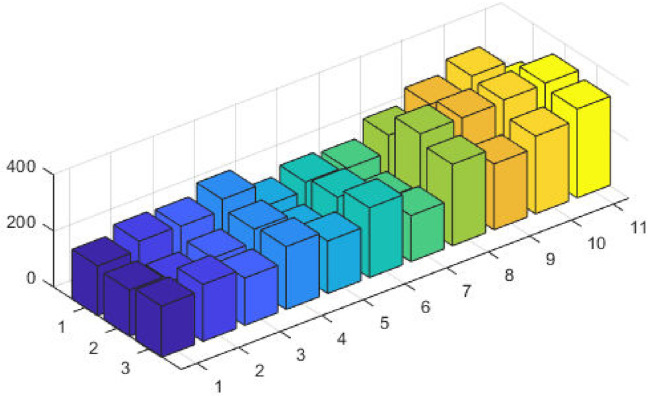
Optimizer power output response against eleven convex machines.

**Fig 10:**
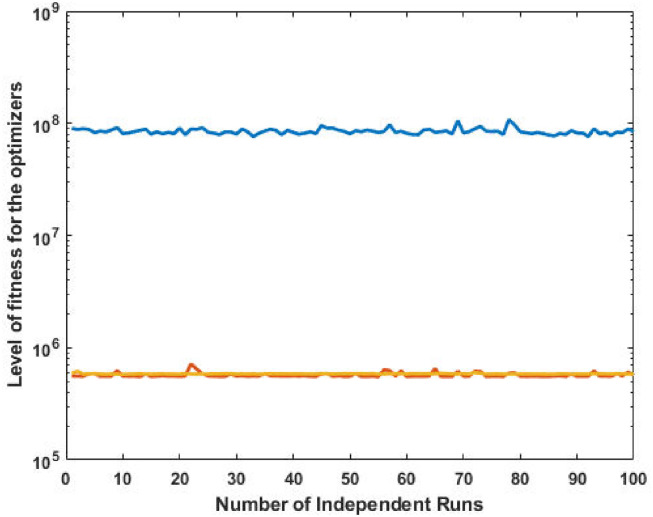
Optimizer fitness level response against eleven convex machines.

The corresponding power values and fitness plots are demonstrated in Figs 9 and 10, respectively. By comparing the cost from [59], our design scheme obtains better (optimal) result with validation of all machine constraints.

### Case Study-VI

**Fig 11:**
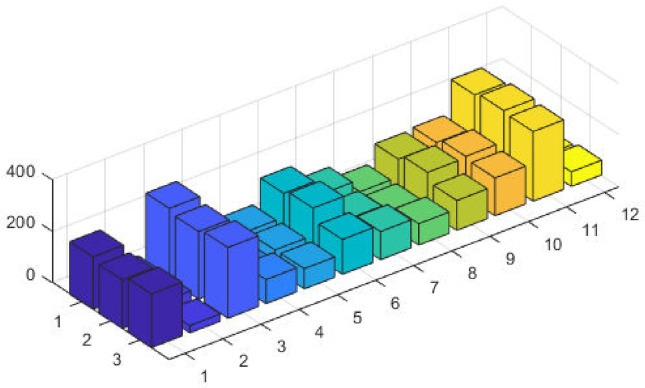
Optimizer power output response against twelve convex machines.

**Fig 12:**
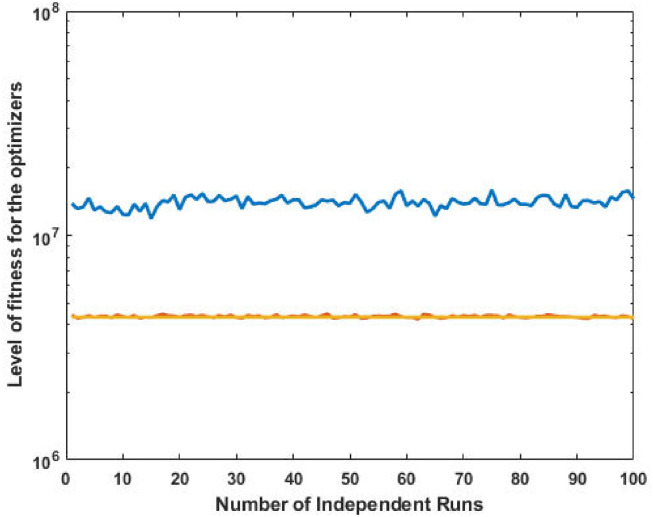
Optimizer fitness level response against twelve convex machine.

This case study is composed of twelve convex machines with total load demand 1600MW with fuel coefficients data as shown in Table 12. The optimal results are shown in Table 13, which shows superiority of the proposed method in terms of computational time and total cost over GA and SQP. GA-SQP returns an optimal cost of 4240100 for Case Study-VI, which is better than the heuristic technique [59], which returns a cost of 43401000. Fig 11 shows the power values in the semi-log scale for increasing the size of the small effects, and the corresponding fitness plots are provided in Fig 12.

**Table 12:** Twelve unit for Case Study-VI.

a	b	c
3.3829	3871.2	117970
35.780	3930.4	38040
2.063	1300.8	21840
0.5492	1364.9	121270
10.606	1143.7	27980
3.034	3073.07	117850
3.230	1631.2	21930
5.062	964.2	88180
1.641	1920.7	59530
1.805	1701.8	65180
0.0078	2212.3	42800
35.780	3930.4	38040

**Table 13:** Optimal generations, cost, and time for Case Study-VI.

Computing Framework	GA	SQP	GA-SQP
CU-1	203.5	188.6	207.9
CU-2	46.6	43.6	28.37
CU-3	278.3	262.5	274.6
CU-4	97.4	67.9	93.3
CU-5	73.1	77.2	78.7
CU-6	166.3	183.1	135.4
CU-7	106.7	88.6	109
CU-8	58.8	59.8	82.7
CU-9	128.7	151.1	116.1
CU-10	136	156.51	146.8
CU-11	264.4	277.2	269.9
CU-12	39.7	43.6	56.8
Total Generation	1600 MW	1600 MW	1600 MW
Best Cost	4321300	4240100	11884000
Best Time	26.6	0.110	0.063

### Case Study-VII

**Fig 13:**
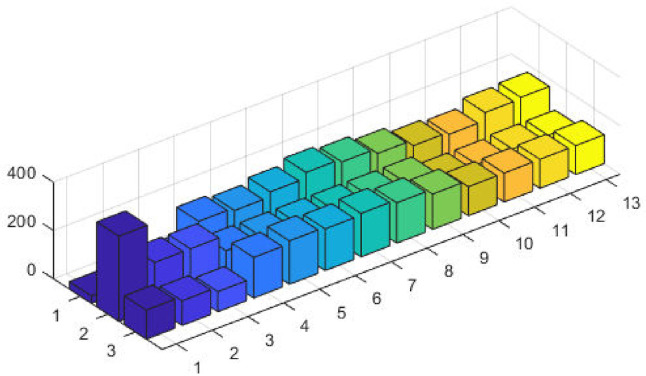
Optimizer power output response against thirteen non-convex machines.

**Fig 14:**
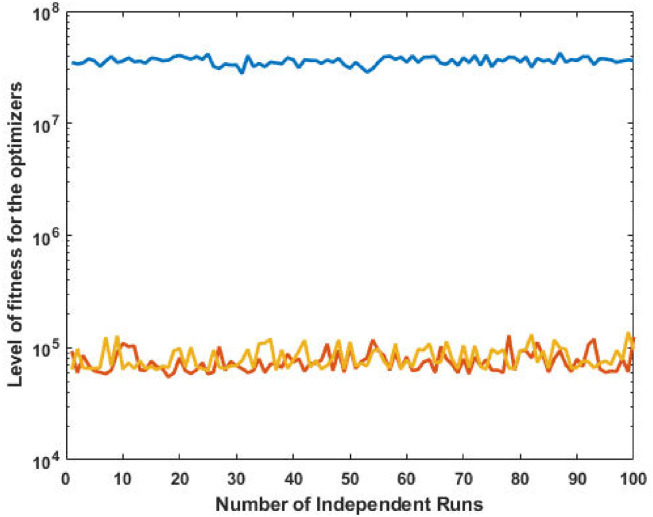
Optimizer fitness level response against thirteen non-convex machines.

The previous cases have not considered the VPLE for a large-scale system; therefore, we consider a thirteen non-convex machines based system with total load demand of 1800MW. For this system, the reference data along with fuel cost and VPLE coefficients can be seen in [70]. By application of the proposed approach, the optimal results are shown in Table 14. Fig 13 shows the power values, and the performance of iterative procedure of fitness is plotted in Fig 14. Our design scheme obtains optimal result while satisfying all constraints appropriately. Note that the results are attained in a small time (0.063s) for the proposed GA-SQP method with optimal results, while validating the total demand and all other constraints. It should be noted that the use of combined approach is interesting due to reduction in computational time and for obtaining better total generation cost.

**Table 14:** Optimal generations, cost, and time for Case Study-VII.

Computing Framework	GA	SQP	GA-SQP
CU-1	31	352.8	120.2
CU-2	36	186.9	102.0
CU-3	57	186.6	83.1
CU-4	169	122.1	168.3
CU-5	157	122.1	179.9
CU-6	178	122.1	172.7
CU-7	201	122.1	179.9
CU-8	188	122.1	167
CU-9	166	122.1	146.4
CU-10	142	81.4	119.9
CU-11	132	81.4	119.9
CU-12	165	88.6	119.9
CU-13	171.727527830706	88.6	119.9
Total Generation	1800 MW	1800 MW	1800 MW
Best Cost	27656000	62393	54785
Best Time	95.3	0.14	0.063

### Case Study-VIII

It composed of fifteen convex machines with total load demand 2630MW. The reference data along with fuel cost and optimal results using the proposed method are shown in [71] and Table 15, respectively.

**Table 15:** Optimal generations, cost, and time for Case Study-VIII.

Computing Framework	GA	SQP	GA-SQP	Computing Framework	GA	SQP	GA-SQP
CU-1	258.8	347.2	314.8	CU-11	118.6	58.7	79.9
CU-2	251.4	347.2	309.2	CU-12	124.2	58.7	79.9
CU-3	157.1	91.1	130	CU-13	135.9	63.7	84.9
CU-4	155.2	91.1	129.9	CU-14	97.3	40.8	54.9
CU-5	293.2	356.9	326.7	CU-15	94.2	40.8	54.9
CU-6	233.9	345.1	309.5	Total Generation	2630MW	2630MW	2630MW
CU-7	210.8	348.3	292.3	Best Cost	69582000	127680	103900
CU-8	165.4	215.1	198.0	Best Time	134.2	0.1389	0.1435
CU-9	179.5	112.2	114.6	-	-	-	-
CU-10	153.9	112.2	149.5	-	-	-	-

**Fig 15:**
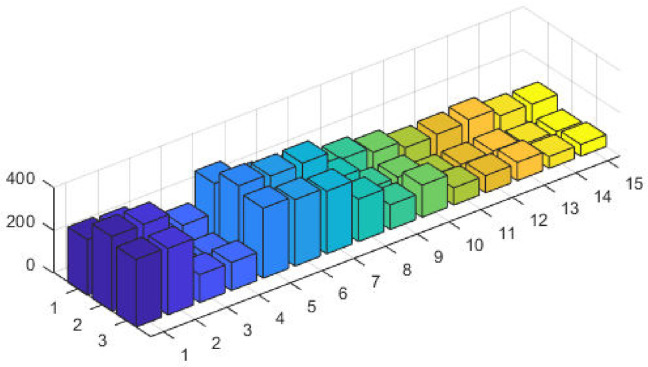
Optimizer power output response against fifteen convex machines.

**Fig 16:**
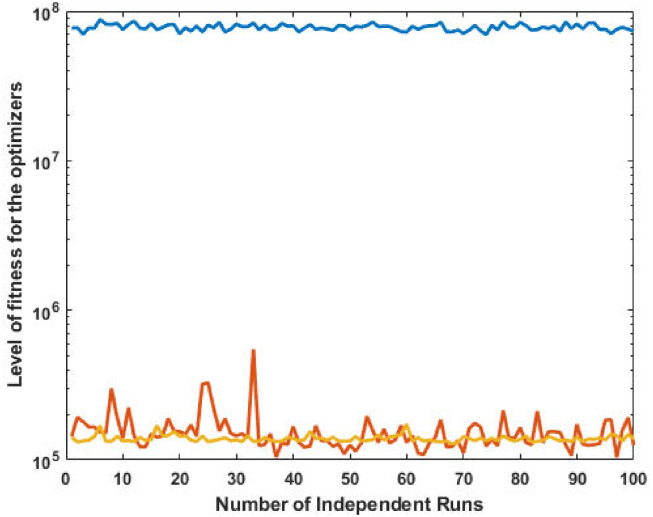
Optimizer fitness level response against fifteen convex machines.

Fig 15 demonstrates the power values. The fitness curves are plotted in Fig 16. It is worth mentioning that our design scheme provides the optimal results in addition to satisfying all the constraints. Hence the proposed method can be used for large-scale problem.

### Case Study-IX

**Fig 17:**
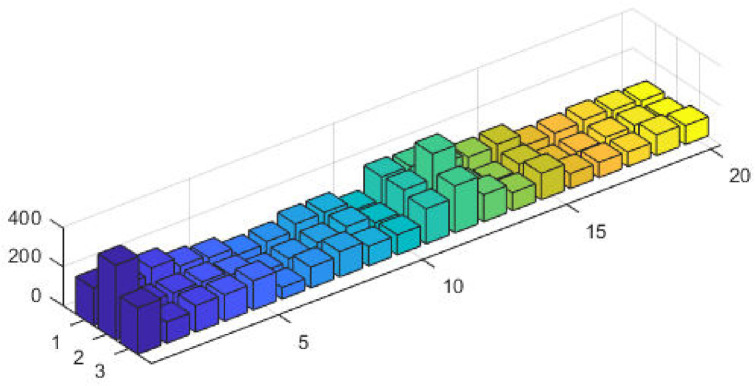
Optimizer power output response against twenty convex machines.

**Fig 18:**
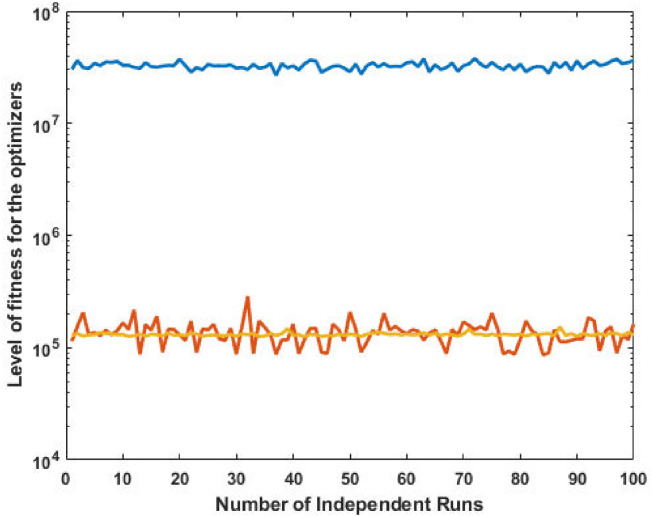
Optimize fitness level response against twenty convex machines.

To consider a further large-scale system, we take twenty convex machines [72] with total load demand 2500MW. The results obtained via GA, SQP, and the proposed GA-SQP approach are provided in Table 16. Figs 17 and 18 demonstrate the corresponding values of generations (in semi-log scale) and fitness function, respectively. It is revealed that our design scheme can be used for large-scale problem such has twenty machines.

**Table 16:** Optimal generations, cost, and time for Case Study-IX.

Computing Framework	GA	SQP	GA-SQP	Computing Framework	GA	SQP	GA-SQP
CU-1	193.1	384.8	314.8	CU-13	145.8	102.6	292.3
CU-2	150.4	128.2	309.2	CU-14	127.9	77.4	198.0
CU-3	182.3	128.2	130	CU-15	136.4	108.5	114.6
CU-4	135.2	128.2	129	CU-16	75.5	51.3	149.5
CU-5	121.2	107.4	326.7	CU-17	81.5	58.7	79.94
CU-6	80.6	61.7	309.5	CU-18	92.6	76.9	79.9
CU-7	90.6	77.1	292.3	CU-19	85.5	81.7	84.9
CU-8	125.4	102.1	198	CU-20	77.1	66.5	54.9
CU-9	127	128.2	114.6	Total Generations	2500 MW	2500 MW	2500 MW
CU-10	80.7	92.6	149.5	Best Time	77.2	0.0872	0.1433
CU-11	197	204.3	326.705773	Best Cost	26768000	124630	86492
CU-12	193.2	332.6	309.5	-	-	-	-

### Case Study-X

As a final simulation, we consider a very-large scale and complicated system to demonstrate the effectiveness and to show the applicability of the proposed method. This system consists of forty non-convex machines with a total load demand of 1800MW. The reference data along with fuel cost and VPLE coefficients is taken from [59]. Table 17 shows the results of the approaches of GA, SQP, and GA-SQP (proposed method).

**Table 17:** Optimal generations, cost, and time for Case Study-X.

Computing Framework	GA	SQP	GA-SQP	Computing Framework	GA	SQP	GA-SQP
CU-1	134.1	92	105.9	CU-21	336.7	466.7	437.8
CU-2	107.7	92	113.9	CU-22	350.4	466.7	459
CU-3	97.7	103.1	118	CU-23	294.4	466.7	447.6
CU-4	146.9	159	174.6	CU-24	312.8	466.7	455.3
CU-5	78.4	82.9	96.9	CU-25	350.3	466.7	461.7
CU-6	170.1	119.7	139.9	CU-26	359	466.7	451.5
CU-7	202.5	246.5	248.4	CU-27	108	110.6	131
CU-8	213.7	253.6	255.8	CU-28	62.5	110.6	132.9
CU-9	215	253.6	267.7	CU-29	103.7	110.6	124.6
CU-10	207.3	252.1	272.4	CU-30	113.8	82.9	96.9
CU-11	141	295.9	267.5	CU-31	161	153.4	158.5
CU-12	209.5	296.7	277.8	CU-32	143.5	153.4	176.77
CU-13	159.2	394.5	351.3	CU-33	161.3	153.4	177.1
CU-14	199.4	394.5	357.7	CU-34	198.9	169	180.6
CU-15	229.3	394.5	371.1	CU-35	124.5	169	173.4
CU-16	202.4	394.5	358.4	CU-36	168.1	169	176.2
CU-17	325.6	421.2	405.7	CU-37	76.9	86	109.9
CU-18	326.9	421.2	409.1	CU-38	104	86	109.9
CU-19	304.6	463.3	454.1	CU-39	94.8	86	108
CU-20	344.8	463.3	442.9	CU-40	284.4	463.3	440.1
Total Generation	-	-	-	Total Generation	10500MW	10500MW	10500MW
Best Cost	-	-	-	Best Cost	8039422.9	3213079	4913442
Best Time	-	-	-	Best Time	844.8	0.797	39.8

**Fig 19:**
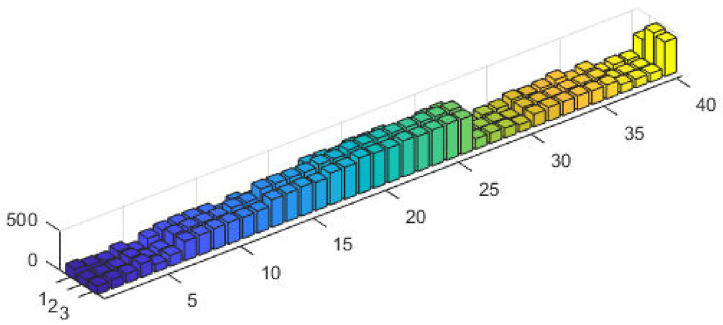
Optimizer power output response against forty non-convex machines.

**Fig 20:**
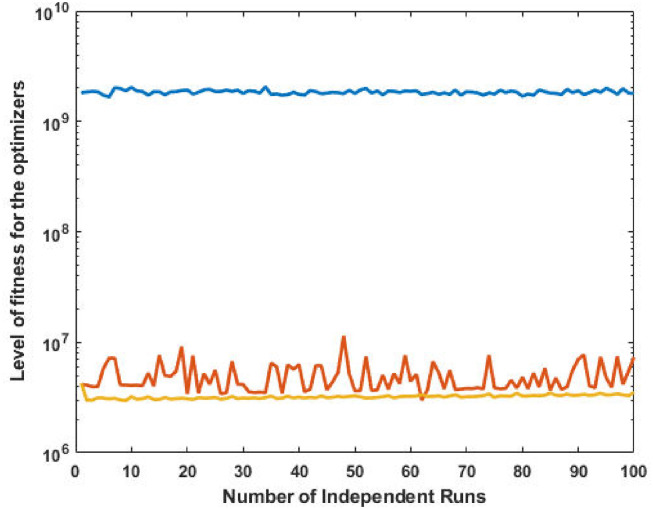
Optimizer fitness level response against forty non-convex machines.

The corresponding power value are shown by Fig 19 at semi-log scale. The resultant fitness curves are also plotted in Fig 20. By comparing cost from [59] our design scheme obtain optimal result while satisfying all constraints appropriately.

Statistical analysis is the process of determining trends, patterns, and relationships through the use of quantitative data, and it is a critical research tool that scientists employ on constrained based objective function. A comprehensive statistical analysis is used to determine the stochastic algorithms’ reliability in terms of the minimum cost (Min cost), the maximum cost (Max cost), the mean cost (Mean cost), and the standard deviation of the cost (STD cost). Not only is the cost value analysed, but also the computational time required for optimization in terms of mean CPU time is also observed. Table 18 demonstrates that the proposed schemes’ results are highly comparable in terms of reliability, effectiveness, economic cost, and computational budget.

**Table 18:** Statistical analysis of different approaches.

Method	Min(cost)	Max (cost)	STD (cost)	Mean (cost)	Min(cost)	Max (cost)	STD (cost)	Mean (cost)	Min(cost)	Max (cost)	STD (cost)	Mean (cost)
Proposed	**CASE STUDY-I**				**CASE STUDY-II**				**CASE STUDY-III**			
GA	8.431e+03	8.871e+03	109.55	8.623e+03	8.219e+03	8.385e+03	32.92	8.274e+03	3.530e+05	5.073e+05	2.854e+04	4.120e+05
SQP	8.596e+03	8.596e+03	3.656e-12	8.596e+03	8.201e+03	8.201e+03	1.096e-11	8.201e+03	3.077e+03	4.578e+03	176.36	3.316e+03
GA-SQP	8.421e+03	8.726e+03	103.18	8.547e+03	8.219e+03	8.249e+03	4.739	8.231e+03	1.711e+03	3.242e+03	427.9	2.402e+03
Proposed	**CASE STUDY-IV**				**CASE STUDY-V**				**CASE STUDY-VI**			
GA	4.143e+07	5.298e+07	2.482e+06	4.696e+07	7.603e+07	1.078e+08	5.318e+06	8.481e+07	1.188e+07	1.596e+07	8.101e+05	1.403e+07
SQP	4.816e+04	7.409e+04	2.912e+03	5.228e+04	5.766e+05	6.160e+05	4.521e+03	5.832e+05	4.321e+06	4.342e+06	2.552e+03	4.326e+06
GA-SQP	4.244e+04	6.678e+04	2.743e+03	4.649e+04	5.518e+05	7.160e+05	2.674e+04	5.701e+05	4.240e+06	4.470e+06	4.877e+04	4.350e+06
Proposed	**CASE STUDY-VII**				**CASE STUDY-VIII**				**CASE STUDY-IX**			
GA	2.765e+07	4.229e+07	2.840e+06	3.594e+07	6.958e+07	8.790e+07	3.871e+06	7.782e+07	2.676e+07	3.778e+07	2.343e+06	3.261e+07
SQP	6.239e+04	1.371e+05	1.911e+04	8.233e+04	1.276e+05	1.723e+05	7.638e+03	1.386e+05	1.246e+05	1.532e+05	4.150e+03	1.310e+05
GA-SQP	5.478e+04	1.288e+05	1.710e+04	7.580e+04	1.039e+05	5.482e+05	5.577e+04	1.558e+05	8.649e+04	2.867e+05	3.415e+04	1.363e+05

The hundred independent iterations were performed for each case study and the optimal generated powers of committed units along with best computational budget and cost are tabulated in each case study via tables. The hundred independent iteration runs in simulations demonstrate that the output comes closer and closer to the optimal cost while maintaining the threshold criteria stated in Eq (11) which further depicts the quality convergence of proposed scheme. Test systems with six-convex machines show how soft restrictions on the objective function enhance the multiple constraints in the structural solution. We tested the proposed soft computing approach viability by using it to various EDP problems. The obtained characteristics costs and time are compared with other state of the art met-heuristic techniques. The optimizer response for handling both categories of test systems are shown in the form of fitness level responses. Its quite evident from tabular data that our proposed soft framework handled all non-convex and contagious constraints efficiently.

There are some recent studies which have considered the convergence rate investigation by considering the rate of change of a factitious energy function [73–75] and [76]. These methods consider the decreasing rate of change of energy based on Lyapunov approach. In future, such methods can be applied for investigating convergence and its rate for an optimization.

## Conclusions

This article discussed the key aspects and concerns of the DEDP problem. Two alternative formulations for the DEDP problem that are convex and non-convex cost functions were studied, and then a novel hybrid GA-SQP was presented to solve the DEDP. The presented scheme can be classified as an artificial intelligence technique, based on the hybrid GA-SQP approach. Due to the capacity of GA-SQP to seek the global optimal solution, the proposed method has been effectively utilized for solving DEDP with non-smooth or non-convex cost functions. It has been observed that hybrid approaches that integrate two or more optimization techniques are more successful in finding the global optimal solution for the DEDP with non-smooth or non-convex cost functions associated with nonlinear constrains. The aim behind the study is to solve the complex optimization problem with constraints for DEDP by considering MFO, RRL, and VPLE. The framework accomplished by coalesce heuristic ability of GA as a global search approach; and then results of that are refined through SQP. Ten highly nonlinear case studies with complex non-convex contiguous constraints were tested. The performance-based indices like computational cost, stability and handling of contiguous constraints validated the precision, dependability and unwavering quality of the proposed framework. While the sensible precision of framework is attained furthermore by acquiring optimal generations with satisfactory cost values. It is recommended to apply these tools to investigate multi-objective DEDP with environmental constraints. By comparing cost from existing papers mentioned in the case studies, our design scheme obtains optimal result while satisfying all constraints appropriately. In future, we can integrate conventional DEDP with environmental cost function along with the renewable energy resources to eliminate the dependency of fossil fuels.

## Supporting information

S1 Dataset (PDF)
