# Supplementary material for: A novel hybrid soft computing optimization framework for dynamic economic dispatch problem of complex non-convex contiguous constrained machines
Source: PLoS One. 2022 Jan 26;17(1):e0261709. doi: 10.1371/journal.pone.0261709 (PMC8791528; doi:10.1371/journal.pone.0261709)
Supplement: S1 Dataset — (PDF) [file pone.0261709.s001.pdf]

Manuscript Reference No: PONE-D-21-20404
Title: A Novel Hybrid Soft Computing Optimization Framework for Dynamic Economic Dispatch Problem of Complex Non-convex Contiguous Constrained Machines

Remaining fuel cost coefficient data for case studies VII, VIII, IX and X

Table 1: **Thirteen unit system for Case Study-VII [1]**

a	b	c	e	f
0.000280	8.100	550	300	0.03500
0.000560	8.100	309	200	0.4200
0.000560	8.100	307	150	0.4200
0.003240	7.740	240	150	0.06300
0.003240	7.740	240	150	0.06300
0.003240	7.740	240	150	0.06300
0.003240	7.740	240	150	0.06300
0.003240	7.740	240	150	0.06300
0.003240	7.740	240	150	0.06300
0.002840	8.600	126	100	0.08400
0.002840	8.600	126	100	0.08400
0.002840	8.600	126	100	0.08400
0.002840	8.600	126	100	0.08400

Table 2: **Fifteen units system for Case Study-VIII [2]**

a	b	c	e	f
0.000299	10.1	671	0	0
0.000183	10.2	574	0	0
0.001126	8.8	374	0	0
0.001126	8.8	374	0	0
0.000205	10.4	461	0	0
0.000301	10.1	630	0	0
0.000364	9.8	548	0	0
0.000338	11.2	227	0	0
0.001203	11.2	173	0	0
0.001203	10.7	175	0	0
0.003586	10.2	186	0	0
0.005513	9.9	230	0	0
0.000371	13.1	225	0	0
0.001921	12.1	309	0	0
0.004447	12.4	323	0	0

Table 3: **Twenty units system for Case Study-IX [3]**

a	b	c	e	f
0.00068	18.19	1000	0	0
0.00071	19.26	970	0	0
0.0065	19.8	600	0	0
0.005	19.1	700	0	0
0.00738	18.1	420	0	0
0.00612	19.26	360	0	0
0.0079	17.14	490	0	0
0.00813	18.92	660	0	0
0.00522	18.27	765	0	0
0.00573	18.92	770	0	0
0.0048	16.69	800	0	0
0.0031	16.76	970	0	0
0.0085	17.36	900	0	0
0.00511	18.7	700	0	0
0.00398	18.7	450	0	0
0.0712	14.26	370	0	0
0.0089	19.14	480	0	0
0.00713	18.92	680	0	0
0.00622	18.47	700	0	0
0.00773	19.79	850	0	0

Table 4: **Forty units system for Case Study-X [4]**

a	b	c	e	f	a	b	c	e	f
0.006900	6.730	94.7050	100	0.08400	0.002980	6.630	785.960	300	0.03500
0.006900	6.730	94.7050	100	0.08400	0.002980	6.630	785.960	300	0.03500
0.0202800	7.070	309.540	100	0.08400	0.002840	6.660	794.530	300	0.03500
0.009420	8.180	369.030	150	0.06300	0.002840	6.660	794.530	300	0.03500
0.01140	5.350	148.890	120	0.07700	0.002770	7.100	801.320	300	0.03500
0.0114200	8.050	222.330	100	0.08400	0.002770	7.100	801.320	300	0.03500
0.003570	8.030	278.710	200	0.04200	0.521240	3.300	1055.10	120	0.07700
0.004920	6.990	391.980	200	0.04200	0.521240	3.300	1055.10	120	0.07700
0.05730	6.600	455.760	200	0.04200	0.521240	3.300	1055.10	120	0.07700
0.006050	12.90	722.820	200	0.04200	0.01140	5.350	148.890	120	0.07700
0.005150	12.90	635.200	200	0.04200	0.001600	6.430	222.920	150	0.6300
0.005690	12.80	654.690	200	0.04200	0.001600	6.430	222.920	150	0.6300
0.004210	12.50	913.400	300	0.03500	0.001600	6.430	222.920	150	0.6300
0.007520	8.840	1760.40	300	0.03500	0.01000	8.950	107.870	200	0.04200
0.007080	9.150	1728.30	300	0.03500	0.01000	8.620	116.580	200	0.04200
0.007080	9.150	1728.30	300	0.03500	0.01000	8.620	116.580	200	0.04200
0.003130	7.970	647.850	300	0.03500	0.01610	5.880	307.450	80	0.09800
0.003130	7.950	649.690	300	0.03500	0.01610	5.880	307.450	80	0.09800
0.003130	7.970	647.830	300	0.03500	0.01610	5.880	307.450	80	0.09800
0.003130	7.970	647.810	300	0.03500	0.003130	7.970	647.830	300	0.03500

References

[1] TH Khoa, PM Vasant, MS Balbir Singh, and VN Dieu. Solving economic dispatch problem with valve-point effects using swarm-based mean–variance mapping optimization (mvmos). *Cogent Engineering*, 2(1):1076983, 2015.

[2] John G Vlachogiannis and Kwang Y Lee. Economic load dispatch—a comparative study on heuristic optimization techniques with an improved coordinated aggregation-based pso. *IEEE Transactions on Power Systems*, 24(2):991–1001, 2009.

[3] M Basu and A Chowdhury. Cuckoo search algorithm for economic dispatch. *Energy*, 60:99–108, 2013.

[4] U Güvenç, YUSUF Sönmez, S Duman, and N Yörükeren. Combined economic and emission dispatch solution using gravitational search algorithm. *Scientia Iranica*, 19(6):1754–1762, 2012.
